# Genetic variants in *MAPT* are associated with Alzheimer's disease blood biomarkers in South Asians from the Longitudinal Aging Study in India‐Diagnostic Assessment of Dementia

**DOI:** 10.1002/alz70855_100340

**Published:** 2025-12-23

**Authors:** Wei Zhao, Zheng Li, Jennifer A Smith, Hasan Abu‐Amara, Yuk Yee Leung, Gerald D. Schellenberg, Li‐San Wang, Priya Moorjani, Eileen M. Crimmins, Bharat Thyagarajan, Masroor Anwar, Peifeng Hu, Sharmistha Dey, Xiang Zhou, Kumarasamy Thangaraj, Aparajit Ballav Dey, Jinkook Lee, Sharon L R Kardia

**Affiliations:** ^1^ University of Michigan Institute for Social Research, Ann Arbor, MI, USA; ^2^ University of Michigan School of Public Health, Ann Arbor, MI, USA; ^3^ Penn Neurodegeneration Genomics Center, Department of Pathology and Laboratory Medicine, Perelman School of Medicine, University of Pennsylvania, Philadelphia, PA, USA; ^4^ Penn Neurodegeneration Genomics Center, Department of Pathology and Laboratory Medicine, University of Pennsylvania Perelman School of Medicine, Philadelphia, PA, USA; ^5^ University of California, Berkeley, Berkeley, CA, USA; ^6^ University of Southern California, Los Angeles, CA, USA; ^7^ University of Minnesota, Minneapolis, MN, USA; ^8^ All India Institute of Medical Sciences, New Delhi, Delhi, India; ^9^ University of California, Los Angeles, Los Angeles, CA, USA; ^10^ Centre for Cellular & Molecular Biology, Hyderabad, India; ^11^ Center for Economic and Social Research, University of Southern California, Los Angeles, CA, USA

## Abstract

**Background:**

Alzheimer's disease (AD) is characterized by abnormal blood levels of specific proteins associated with amyloid plaques, tau pathology, and neurodegeneration, which are considered biomarkers of AD. To better understand the genetic architecture of AD in South Asians, we assessed the association between single nucleotide polymorphisms (SNPs) previously associated with AD and AD biomarkers with blood‐level measures of seven AD biomarkers in 2,547 South Asians from the Harmonized Diagnostic Assessment of Dementia for the Longitudinal Aging Study in India (LASI‐DAD).

**Method:**

We performed SNP‐based association analysis between 152 AD‐ and 9 AD biomarker‐associated SNPs with 7 AD biomarkers, including amyloid beta 40 (Ab40), amyloid beta 42 (Ab42), Ab42/Ab40 ratio, total tau (tTau), tau phosphorylated at threonine 181 (pTau181), glial fibrillary acidic protein (GFAP), and neurofilament light chain (NfL). The model adjusted for age, sex, the first 10 genetic principal components (PCs), and incorporated the genetic relationship matrix and plate as random effects. Significance was declared at Benjamini‐Hochberg FDR q<0.1. Identified loci were followed up with haplotype analysis to understand the potential driving factors.

**Result:**

Among 152 AD‐associated SNPs, rs199515_C (*WNT3/MAPT*) was associated with tTau in the expected direction (beta=0.21, *p* = 0.00052), whereas rs2718058_A (*NME8*) was associated with pTau181 in the unexpected direction (beta=0.13, *p* = 0.00057). Among the 9 biomarker‐associated SNPs, rs242557_A (*MAPT*) was associated with tTau (beta=‐0.139, *p* = 2.3e‐08) and pTau181 (beta=0.099, *p* = 1.4e‐04), and rs429358_C (*APOE‐e4*) was associated with Ab42/Ab40 ratio (beta=‐0.141, *p* = 0.0012). Follow‐up analysis in *MAPT*, the gene that encodes the tau protein, identified a significant association between H1/H2 haplotype and tTau (global‐p=0.00205). Specifically, H1j (*p* = 0.0015), H2 (*p* = 0.027), H1r (*p* = 0.031), and H1b (*p* = 0.041) were associated with lower levels of tTau, whereas H1c (*p* = 0.04) and H1o (*p* = 0.012) were associated with higher levels. Moreover, *APOE*‐*e4* stratified analysis detected significant H1j‐tTau (score=‐3.61, *p* = 0.00031) and H1c‐pTau181 (score=2.55, *p* = 0.01) associations in *e4* carriers only.

**Conclusion:**

The analysis highlights a critical role of *MAPT* in tau pathology in South Asians. The observed association does not seem to be fully driven by known risk haplotypes. It also provides evidence to suggest modification by *APOE‐e4*. Further investigation of *MAPT* haplotype structure in this population is warranted.

